# Slow-wave sleep in the TPJ is linked to individual differences in implicit bias

**DOI:** 10.1038/s41598-025-27777-1

**Published:** 2025-11-24

**Authors:** Mirjam Studler, Lorena R. R. Gianotti, Marina Wunderlin, Daria Knoch

**Affiliations:** 1 Department of Social Neuroscience and Social Psychology, Institute of Psychology, University of Bern, Fabrikstrasse 8, 3012 Bern, Switzerland; 2 University Hospital of Old Age Psychiatry and Psychotherapy, University of Bern, Bolligenstrasse 111, 3000 Bern 60, Switzerland

**Keywords:** Deep sleep, Implicit bias, Sleep slow-wave activity, Social cognition, Source-localization, Temporo-parietal junction, Sleep, Slow-wave sleep

## Abstract

Implicit biases can profoundly impact social interactions and decision-making processes. Here, we investigate whether the heterogeneity of implicit racial bias can be explained by interindividual differences in the intracortical distribution of slow-wave activity (SWA), a marker of sleep depth. Using high-density EEG, we recorded sleep data from 52 healthy adults (27 females) during a typical night’s sleep in their homes. Implicit racial bias was measured using the race Implicit Association Test (IAT). Whole-brain corrected source localization analysis identified a significant association between higher relative SWA current density in the left temporoparietal junction (TPJ) and lower levels of implicit racial bias. The TPJ plays a crucial role in social cognitive processes such as mentalizing - the process of understanding others’ mental states. We propose that deeper sleep in this region, as indicated by higher SWA current density, may reflect higher inherent mentalizing abilities, leading to reduced implicit racial bias. Applying a novel approach to investigate the link between sleep and social functioning, our data suggests that local aspects of sleep could explain inter-individual differences in social cognition.

## Introduction

People often hold unconscious prejudices against individuals with different skin colors. Although many individuals consciously reject having negative biases toward others, these biases often become evident through implicit measures^1^. Such implicit biases are presumably one of the main reasons for the persistence of discrimination [e.g.,^2,3^]. They can influence social behavior in various ways – from impression formation to concrete behavior. Implicit racial bias underlies vast inter-individual variability [e.g.,^4,5^]. Despite the substantial variability and influence on behavior and perception, only a few studies have investigated dispositional determinants of implicit racial bias [e.g.,^6,7^]. An ideal approach to illuminate sources of inter-individual variability is the investigation of neural traits, which are dispositional brain-based characteristics^8,9^. Neural traits represent an objective and stable measure for identifying sources of behavioral heterogeneity, free from personal biases^8^. It has recently been shown that trait-like characteristics of sleep can be used as a neural trait to explain variance in diverse behavioral phenotypes^10–12^. Here, we set out to explain individual differences in implicit racial bias through such a neural trait as measurable during sleep.

Evidence links sleep to implicit racial bias. In one study, a three-week sleep restriction protocol increased implicit racial bias compared to three weeks of normal sleep^13^. Relatedly, targeted memory reactivation during sleep has been reported to temporarily reduce implicit bias^14^. While these findings hint towards a potential protective role of sleep against implicit bias^13^, an open question is how sleep affects implicit bias without experimental manipulation of sleep duration, and whether inter-individual differences in sleep characteristics can explain variance in implicit racial bias. To date, no study has examined the relationship between implicit racial bias and dynamics of the undisturbed, sleeping brain, even though understanding its role is crucial for uncovering the mechanisms through which sleep links to implicit racial bias. In our study, we investigated slow-wave activity (SWA) during sleep. SWA is a hallmark of deep sleep and a proxy for sleep depth. The topographical distribution of SWA is stable within individuals, but shows distinct differences between individuals, thus serving as an electrophysiological “fingerprint” unique to each sleeping person^15–18^. As such, it can be considered a neural trait, making it an ideal candidate for capturing individual differences in implicit bias. Moreover, previous work has shown that regional SWA predicts inter-individual differences in other domains such as risk preferences^10^ and prosocial preferences^19,20^. This suggests that trait-like SWA may systematically relate to stable social-cognitive tendencies and may also extend to implicit bias.

We assessed the intracortical distribution of relative SWA under normal, undisturbed sleep of good sleepers (habit of 7–8 h / night) and linked these individual differences to individual differences in implicit racial bias. We applied an Implicit Associations Task (IAT^4^), which is widely used to identify individual differences in implicit bias [e.g.,^4^]. As this is the first study relating sleep characteristics to implicit racial bias, we do not make concrete predictions about which brain areas might be involved but rather use an explorative, whole-brain corrected source localization approach. However, based on previous literature on brain regions involved in implicit bias, we could tentatively expect relative SWA differences in areas such as the prefrontal cortex (PFC), the temporoparietal junction (TPJ), the insula, the anterior cingulate cortex (ACC), or the orbitofrontal cortex (OFC)^9,21–25^. In sum, the present study elucidates how differences in the intracortical distribution of relative SWA under normal, experimentally unmanipulated conditions are linked to the heterogeneity of implicit racial bias.

## Materials and methods

### Participants

We performed an a priori power analysis and determined the sample size necessary to detect significant correlations with 80% power (alpha = 0.005) using G*Power 3.1.9.7 [F-tests, linear multiple regression;^26^]. Drawing from our previous sleep study on neural traits and risk preferences^10^, as well as other studies examining neural traits in relation to intergroup bias^9,27,28^, we assumed a medium to large effect size of *f* ^*2*^ = 0.25. This power analysis indicated a recommended sample size of 58 participants. As our sleep EEG recordings were conducted at the participants’ homes and without continuous supervision of an experimenter, we anticipated potential dropouts due to technical issues. To account for this, we collected data from 62 healthy, right-handed adults recruited at the University of Bern. Ten participants were excluded due to noncompliance with the study protocol (*n* = 2), insufficient quality of EEG data (*n* = 6), or interruption of the whole-night EEG recordings (*n* = 2). This resulted in a final sample of 52 participants for analyses (mean age = 21.5 years old, SD age = 2.0, 27 females). We specifically recruited a homogenous sample of good sleepers to maximize the chance of measuring people’s normal, characteristic sleep patterns (trait), rather than sleep patterns underlying temporary conditions (state). Consequently, participants were screened to meet the following inclusion criteria: good general sleep quality indexed by a Pittsburgh Sleep Quality Index (PSQI) below 5^29^, habitual sleep duration of 7–8 h per night with regular bedtimes, a typical level of daytime sleepiness expressed by an Epworth Sleepiness Scale (ESS) score below 10^30^, a neutral or moderate chronotype between 2 and 7, as measured by the Munich Chronotype Questionnaire (MCTQ)^31^, no travelling across more than two time zones within the last 30 days preceding the experiment and no history of sleep disorders. Moreover, additional criteria included right-handedness^32^, no history of neurological, psychiatric, or substance use disorder, no regular medication intake, none or moderate caffeine, nicotine, and alcohol consumption, as well as normal body weight. To mitigate the potential impact of the menstrual cycle phase on sleep^33^, the forward counting method was employed. Women with natural menstrual cycles were not scheduled for the experiment during their estimated fertile days or the first two days of menstruation, while women using hormonal contraception were not scheduled during pill-free intervals. Participants provided written informed consent and were compensated with 155 Swiss Francs (USD 177.00) for participation. This study was approved by the local ethics committee of the Faculty of Human Sciences (University of Bern, Switzerland) and was part of a larger research project. All methods were performed in accordance with relevant ethical guidelines and regulations.

### Procedure

**Fig. 1:**
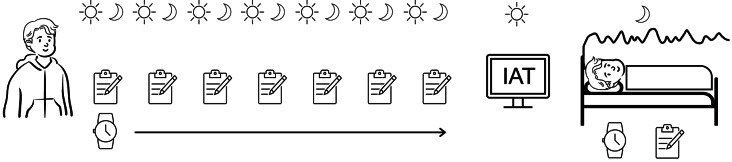
Study design. Participants were instructed to follow a regular sleep-wake schedule for one week prior to the experiment. To objectively track their adherence, they were provided with an actigraph. Throughout this week, participants also filled out sleep and consumption diaries. On the day of the experiment, participants arrived at the laboratory to complete the IAT. Following the IAT, they were equipped with a portable high-density EEG system and sent home, where their sleep EEG was recorded during the following night.

Participants were scheduled for a first laboratory visit one week before the experiment (as depicted in Fig. 1, where the study design is illustrated). During this visit, they were instructed to maintain a regular sleep-wake rhythm that was aligned with their habitual bedtimes and a sleep duration of 7–8 h, as well as to avoid naps during the day for the entire week preceding the experiment. Participants were moreover advised to limit their caffeine consumption to two units per day (equivalent to one cup of coffee per unit) and to restrict alcohol intake to one standard drink per day (one standard drink corresponds to one 350 ml beer = 10 g ethanol). Smokers were instructed to maintain their regular nicotine consumption. To monitor participants’ adherence to the study protocol, sleep-wake rhythms were measured objectively through motion detection. Therefore, participants were provided with a triaxial accelerometer (GENEActiv, Activinsights) to wear on their non-dominant hand. Single-use straps ensured continuous wear throughout the week of protocol. Compliance with the study protocol was further verified using sleep and consumption diaries. Participants were also equipped with a chest harness and a mock amplifier to mimic the experience of wearing the portable high-density EEG system. They were requested to sleep with this setup to determine the best amplifier position for the experimental night with the EEG system.

On the day of the experiment, participants arrived at the behavioral laboratory at 4.30 p.m., where they first completed the IAT. Following the behavioral data collection, they were fitted with the portable high-density EEG system and then sent home to continue their habitual evening routine. Just before bedtime, as determined by their habitual sleep schedule, experimenters visited participants at their homes to verify, and if necessary, adjust electrode impedances to ensure good EEG signal quality and to start the recording. The following morning, participants removed the EEG system themselves and brought it back to the laboratory.

### Measurement of implicit racial bias

We measured participant’s implicit racial bias using the race Implicit Association Test (IAT), which assesses the strength of a person’s automatic associations between racial categories and evaluative terms^4^.

Participants are shown images representing two racial categories (“white” and “black” faces) and words representing two evaluative categories (“positive” and “negative” words). The stimuli appeared in the middle of a PC screen and participants were asked to classify them as quickly and accurately as possible into the four categories by pressing one of two response keys with their left and right index fingers, respectively. The rules of category-response assignments changed from block to block, and the categories were presented throughout the block in the upper left- and right-hand corner of the screen. The IAT contained seven blocks with 190 trials in total. In the first two blocks (20 trials each), participants learned to classify positive vs. negative words and white vs. black faces, respectively. In the third and fourth “congruent” blocks (60 trials in total), participants had to press one key when positive words or white faces appeared, while they had to press another key when negative words or black faces were shown. In the fifth block, assignments for positive and negative words were reversed (30 practice trials), so that white faces and negative words shared the same response key, while black faces and positive words shared another response key in the sixth and seventh “incongruent” blocks (60 trials in total). Please note that we use the terms “congruent” and “incongruent” from the perspective of white participants (51 of the 52 participants). In each trial, the stimuli remained on the screen until the response, followed by a screen where only the category labels were shown with a randomly jittered duration ranging from 300 to 500 ms. Incorrect responses were followed by a red “X” and could not be corrected. The stimuli were sourced from Project Implicit^34^ and were presented via OpenSesame^35^.

### Analysis of IAT data

We calculated the mean response time (RT) of all correct trials for each participant. IAT D-Scores were then calculated according to the conventional revised scoring algorithm^36^. This score is calculated by dividing the RT difference between incongruent and congruent trials by the pooled standard deviation across these trials, thereby adjusting for each participant’s variability in latency. IAT D-Scores range from − 2 to + 2, where a value of zero indicates no implicit racial bias. Positive scores indicate a strong association between white race and positive valence and/or a strong association between black race and negative valence. As we were interested in the strength of implicit racial bias in both directions (i.e. bias towards white race and bias towards black race), we calculated the absolute IAT D-Scores for further analyses.

### Sleep EEG recording and preprocessing

Whole-night sleep data were recorded using a high-density portable EEG system (LiveAmp 64, Brain Products) with 64 channels, including three electrooculogram electrodes and 2 submental electromyogram electrodes. Two additional channels served as recording reference (Cz) and as ground (AFz). The EEG signal was sampled at 500 Hz (third-order low pass filter at 131 Hz), and impedances were kept below 25 kΩ. The recorded signals were offline band-pass filtered between 0.5 and 40 Hz. Sleep stages were defined for 30-second epochs according to standard scoring criteria^37^. Objective sleep quality and sleep architecture were assessed by determining total sleep time, sleep efficiency (proportion of total time in bed spent asleep), wake after sleep onset (WASO, length of periods of wakefulness occurring after sleep onset), and percentage of the total sleep time spent in sleep stages N1, N2, N3, and in non-rapid eye movement (REM) sleep. Subsequently, data from seven channels solely used for sleep scoring (two electromyograms, three electrooculograms, and two mastoid channels) were excluded, leaving 59 electrodes for further analysis. Bad channels were identified based on visual inspection of time-frequency plots and spectrograms and excluded if problematic at any time of the entire night. Signals from the excluded channels were interpolated using spherical linear interpolation^38^, and the signal at each channel was then re-referenced to the average of all channels. Epochs with artifacts were excluded semi-automatically when power in the frequency bands 0.8–4.6 Hz and 20–40 Hz exceeded a moving-average threshold^39^. A Fast Furrier Transformation (using a Hanning window) was applied to all artifact-free 30s epochs in sleep stages N2 and N3 to calculate the power spectra for slow-wave frequencies between 0.8 and 4.6 Hz^40–42^. The spectra for each channel were averaged over all epochs for each participant.

### SWA source localization

Standardized low-resolution brain electromagnetic tomography [sLORETA;^43^] was used to estimate the intracerebral electrical sources that generated the scalp-recorded activity. sLORETA computes electrical neural activity as current density (A/m2) without assuming a predefined number of active sources. The sLORETA solution space consists of 6239 voxels (voxel size: 5 × 5 × 5 mm) and is restricted to cortical gray matter and hippocampi, as defined by the digitized Montreal Neurological Institute probability atlas. The sLORETA algorithm has been used in sleep EEG studies^44–46^ and has been applied to estimate the cortical localization of NREM sleep sources^11,47–49^. The transformation matrix with the signal-to-noise ratio set to 10 was selected using the manual regularization method in the sLORETA software. To reduce confounds with no regional specificity for each participant, sLORETA images were normalized to a total current density of one, yielding sLORETA images of relative SWA current density. These images were then log-transformed before statistical analyses.

### Statistical analysis

A whole-brain voxel-wise correlation approach was taken to identify regions where relative SWA current density correlate with the absolute IAT D-Score. We used voxel-by-voxel partial Pearson correlation analyses to statistically control for the putative influence of total sleep time on the relationship between relative SWA current density and the absolute IAT D-Score. The correction for multiple testing was incorporated using the nonparametric permutation test described in Nichols and Holmes^50^. In detail, 5000 permutations were run in order to estimate the empirical probability distributions. The statistical r-images were then thresholded at the corresponding critical probability threshold (corrected for multiple comparisons at *p* < 0.05), and voxels with statistical values exceeding this threshold were considered as significant. In a next step, the voxel with the strongest correlation (peak voxel) was used for the constructions of a spherical region of interest (ROI; radius: 15 mm around the peak voxel) for the region that displayed significant, whole-brain corrected correlations. Normalized SWA mean current density within the ROI was calculated and used for statistical analyses and for visualization. We conducted laterality analyses to formally test whether findings were specific to one hemisphere. The homologous contralateral region was identified (by reversing the x-coordinates), and the averaged current density was calculated across all voxels within the identified cluster of interest. We then computed a Pearson correlation between the contralateral region and the absolute IAT D-Score. Finally, the two correlations, one for each hemisphere, were compared using Meng’s test^51^ for comparing dependent correlation coefficients.

For further analysis, the partial Pearson correlations approach between relative SWA current density within the ROI and the absolute IAT D-Score was repeated for each individual sleep cycle, controlling for the duration of the specific sleep cycle. Sleep cycles were defined according to the adapted criteria of Feinberg and Floyd^52–54^. Analog as above, a global normalization and log-transformation of the sLORETA images was carried out for each sleep cycle prior to subsequent statistical analyses.

## Results

### Behavioral results and sleep parameters

**Fig. 2:**
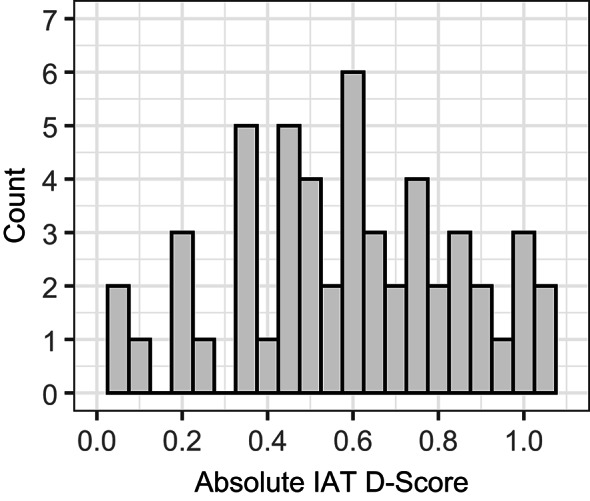
Histogram illustrating the distribution of absolute IAT D-scores. According to established psychological standards for effect sizes, absolute IAT D-Scores can be categorized as follows^55^: Scores less than or equal to 0.20 indicate little or no bias, scores between 0.20 and 0.49 indicate weak bias, scores between 0.49 and 0.74 indicate moderate bias, and scores greater than 0.74 indicate strong bias.

To assess overall implicit racial bias, we examined participants’ performance across conditions. Accuracy rates were 92% for the incongruent condition and 97% for the congruent condition [incongruent errors: *M* = 4.52, *SD* = 3.00; congruent errors: *M* = 1.89, *SD* = 1.71; *t*(61) = 7.72, *p* < 0.001, *η²* = 0.49]. Reaction times (RTs) were significantly longer in incongruent trials (*M* = 844.51 ms, *SD* = 157.41 ms) than in congruent trials [*M* = 673.84 ms, *SD* = 100.61 ms; *t*(61) = 9.90, *p* < 0.001, *η²* = 0.62]. Participants showed considerable individual variability in their implicit racial bias, with absolute IAT D-Scores ranging from 0.03 to 1.05 (*M* = 0.58,* SD* = 0.27; see Fig. 2). Sleep parameters fell within the expected range for individuals of this age group (Table 1). Moreover, objective sleep efficiency measured by actigraphy did not significantly differ between the nights before the EEG measurement and the EEG night itself (92.0% vs. 93.1%).

**Table 1:** Mean with 95% CIs for sleep parameters (total sleep time, sleep efficiency, wake after sleep onset, and duration of sleep stages) for all participants (*N* = 52).

	Total sleep time [min]	Sleep efficiency [%]	Wake after sleep onset [min]	Duration of sleep stages (% of total sleep time)			
				N1	N2	N3	REM
Mean 95% CIs	435.5 427.7-443.4	93.0 92.2–93.9	22.0 18.6–25.4	7.8 6.9–8.8	46.4 44.8–48.0	24.5 22.9–26.0	21.3 20.2–22.4

Sleep efficiency refers to the proportion of time asleep while lying in bed.

### Brain results

**Fig. 3:**
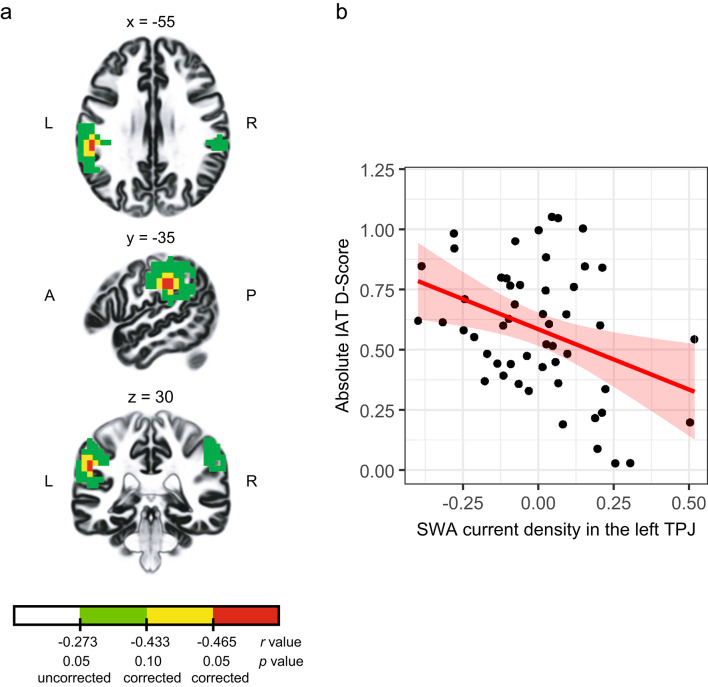
Relationship between relative SWA current density and implicit racial bias corrected for total sleep time. (**a**) Relationship between relative SWA current density in the bilateral TPJ and implicit racial bias. Colors indicate locations of the voxels that showed significant correlations (red p < 0.05, whole-brain corrected, yellow p < 0.10, whole-brain corrected and green p < 0.05, uncorrected). A, anterior; P, posterior; L, left; R, right. (**b**) Scatterplot of the negative association between relative SWA current density within 15-mm spheres around the peak voxel and racial implicit bias controlled for total sleep time (including regression line and 95% confidence interval). We found a significant negative association (*p* = 0.001, *r* = -0.45, *R*^*2*^ = 0.20) between relative SWA current density and implicit racial bias .

As shown above, participants vastly differed in their implicit racial bias. Accordingly, to investigate whether deep sleep neural signatures could explain individual differences in implicit racial bias, we conducted whole-brain correlation analyses using the absolute IAT D-Score as the dependent variable while controlling for total sleep time. Using sLORETA as a source localization technique to estimate intra-cerebral sources underlying scalp-recorded sleep EEG, we found that a whole-brain corrected cluster of voxels in the left TPJ showed significant negative correlations between current density and the absolute IAT D-Score (Fig. 3; cluster size = 500 mm3; MNI coordinates peak voxel: x = -55, y = -35, z = 30, Brodmann area 40). Correlation analyses conducted with the ROI (spheres of 15 mm radius around the peak voxel) revealed negative correlation coefficients of -0.45 (*p* = 0.001, *R*^*2*^ = 0.20). Importantly, controlling for the time participants spent in deep sleep (i.e., sleep stages N2 and N3) did not alter the significant association between relative SWA in the left TPJ and absolute IAT D-Scores (*p* = 0.001, *r* = -0.44, *R*^*2*^ = 0.19), indicating that this relationship is independent of the amount of deep sleep. Additionally, adjusting for participants’ age and gender also did not impact the observed correlation (*p* < 0.001, *r* = -0.41, *R*^*2*^ = 0.17). Meng’s test for dependent correlations did not confirm the laterality effect: The correlation between the absolute IAT D-Score and current density in left TPJ was not significantly different from the correlation between the absolute IAT D-Score and current density in the homologous right TPJ (*Z* = 1.16, *p* = 0.12). This result is confirmed by the fact that lowering the threshold to *r* = − 0.273 (corresponding to an uncorrected p-value of 5%) reveals a second cluster in the region of the right TPJ. As shown in Fig. 3, it is interesting to note that even with a lower threshold, our results are specific to the TPJ, bilaterally.

**Fig. 4:**
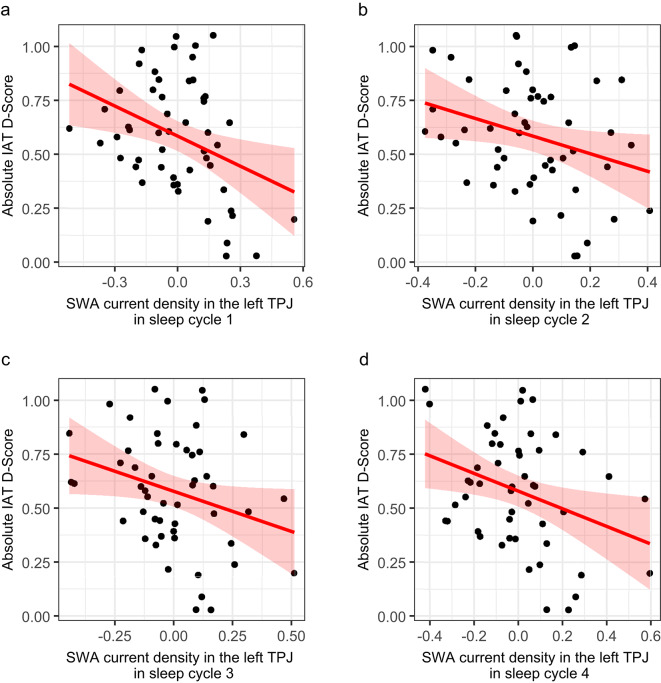
Scatterplots of the negative relationships between relative SWA current density within 15-mm spheres around the peak voxel within the left TPJ and implicit racial bias (including regression line and 95% confidence interval) for (**a**) sleep cycle 1 (*r* = -0.46, *p* = 0.001, *R*^*2*^ = 0.21), (**b**) sleep cycle 2 (*r* = -0.32, *p* = 0.024, *R*^*2*^ = 0.10), (**c**) sleep cycle 3 (*r* = -0.29, *p* = 0.04, *R*^*2*^ = 0.08), and (**d**) sleep cycle 4 (*r* = -0.35, *p* = 0.014, *R*^*2*^ = 0.12), all controlled for total sleep time within the specific sleep cycle.

Levels of SWA exhibit a decline throughout a night’s sleep^56^. Recognizing that the rate of decline differs across cortical regions, we conducted additional analyses to investigate overnight dynamics. Specifically, we correlated the averaged relative SWA current density within 15-mm spheres centered on MNI coordinates of the peak voxel with implicit racial bias for each individual sleep cycle and controlled for the total sleep time within each cycle. As some participants did not have had a fifth sleep cycle, our analysis focused on the first four cycles. As represented in Fig. 4, the results show a similar pattern for all sleep cycles over the whole night. In an additional analysis, we also excluded data from the first sleep cycle to ensure that SWA in the first cycle did not drive our main result. That is, we correlated relative SWA current density within 15-mm spheres centered on the peak voxel pooled together across the second, third, and fourth sleep cycles with implicit racial bias and controlled for total sleep time across these sleep cycles, yielding a significant negative association (*p* = 0.007, *r *= -0.37, *R*^*2*^ = 0.14) again.

## Discussion

There has been increased recognition of the link between sleep and social cognition. Research on sleep deprivation demonstrated that sleep loss adversely affects social cognition^57–62^. Specifically, one study showed that sleep restriction elevates implicit racial bias^13^. While these studies reveal the negative impact of inadequate sleep on social cognitive abilities, including implicit racial bias, they provide no understanding of the underlying mechanisms within sleep or how sleep links to inter-individual variations in social cognition. To close this gap, we directly examined the sleeping brain under normal, undisturbed sleep conditions. In self-reported good sleepers, we examined whether the intracortical distribution of relative SWA current density during natural sleep could explain individual differences in implicit racial bias. Our whole-brain corrected source localization approach revealed a significant negative association between relative SWA in the left TPJ and implicit racial bias. Specifically, higher relative SWA in the left TPJ was associated with lower levels of implicit racial bias.

During wakefulness, the TPJ has been widely recognized for its critical role in social cognition processes such as mentalizing^63^, empathy^64^, perspective-taking^65^ and theory of mind^66^. These TPJ-related functions share a common focus on observing and interpreting other people’s mental states, including their beliefs, needs and intentions^63,67,68^. The importance of the TPJ in social cognition relating to implicit racial bias has previously been discussed^23^. One study demonstrated that a mentalizing intervention increased activity in the TPJ, with the magnitude of this increase predicting the reductions in implicit bias^69^. The authors suggested that the TPJ links to implicit bias through its role in mentalizing, i.e. that increased engagement in understanding others’ mental states – through increased TPJ activity - can lead to reductions in implicit bias^69^. The idea that the TPJ is involved in implicit bias is further supported by studies showing that perspective-taking – a key function of the TPJ – has been linked to reduced implicit racial bias [^70–73^; but see^74^].

Here, we examined the entire brain during sleep, and the TPJ stood out as the only region that was linked to implicit racial bias. Remarkably, a study investigating how EEG-based neural traits during wakefulness relate to implicit intergroup bias also selectively identified activity in the TPJ as a key factor^9^. Specifically, implicit bias, as measured via the IAT, was associated with resting-state EEG activity in the right TPJ during wakefulness. In our study, we similarly found that the IAT was associated with EEG activity in the TPJ during sleep. Although we found more pronounced effects in the left TPJ and weaker associations in the right TPJ, subsequent lateralization analyses using Meng’s test revealed no significant differences between the two hemispheres. This suggests that TPJ involvement in social cognition is not hemisphere-specific but rather bilateral, in line with previous research^63,75,76^. The similarity of the resting-state EEG finding and the results of the present study provides support for the assumption that differences in TPJ activity—whether during wakefulness or during sleep—can explain inter-individual differences in implicit bias. In line with these findings, a study discussing the role of the TPJ in behavioral intergroup bias found that differences in both TPJ white matter integrity and TPJ-dmPFC connectivity strength explain differences in behavioral intergroup bias^28^. As increased white-matter integrity and connectivity strength are thought to enhance the functioning of the associated brain regions^77^, these findings imply that a well-functioning TPJ, indicative of better socio-cognitive processing, links to reduced bias.

In the present study, we found a significant negative association between relative SWA in the TPJ and implicit racial bias. SWA, as a key indicator of sleep depth, is fundamental for understanding the significance of sleep on brain function. High levels of SWA have been associated with higher restorative mechanisms^78,79^, promoting increased cognitive functioning^80,81^. We speculate that higher SWA in the left TPJ may reflect a person’s predisposition for better local restorative processes, thereby allowing for increased TPJ functionality. We suggest that higher SWA in the left TPJ may support an individual’s capacity for mentalizing and/or perspective-taking, which is then mirrored by a decreased inclination toward implicit racial bias. A similar line of research has recently emerged, with two studies using a neural trait approach during sleep to explain diverse behavioral phenotypes. These studies also emphasized how variations in local SWA may reflect differences in sleep-related neural restorative functions, which shape distinct variations of social cognition and behavioral preferences^10,11^. Critically, the relationship between relative SWA and implicit racial bias remained robust in the current study, even after accounting for the time participants spent in deep sleep. This suggests that local restorative processes occur independently of deep sleep quantity, which highlights the crucial role of sleep quality over quantity in shaping social cognition capacity.

In the present study, we used the intracortical distribution of relative SWA as a neural trait to explain differences in implicit racial bias. Several factors reinforce our confidence that SWA, as measured in our study, reflects stable, trait-like characteristics rather than temporal fluctuations. In the seven days leading up to the experiment, participants kept sleep and consumption diaries and wore actigraphy devices to verify compliance with the study protocol, including a regular sleep-wake rhythm, 7–8 h of sleep per night, and no daytime napping. This approach was designed to minimize potential state-related effects. Additionally, since absolute SWA (i.e. without normalization) is subject to day-to-day variations, we normalized SWA, by focusing on its relative intracortical distribution. Moreover, since sleep pressure and absolute SWA levels are highest in the first sleep cycle in the beginning of the night^82,83^, we conducted separate analyses for the individual sleep cycles and found that the association between relative SWA in the TPJ and implicit racial bias was not confined to the first sleep cycle, but remained consistent over all sleep cycles across the night.

In conclusion, our study provides evidence linking individual variations of relative SWA in the TPJ during normal sleep to individual differences in implicit racial bias. The findings highlight the crucial role of sleep quality in shaping social cognition capacity, suggesting that local aspects of sleep may account for intra-individual differences in social cognition. Specifically, we suggest a neural basis for how sleep patterns contribute to variations in implicit racial bias, emphasizing the role of sleep depth in the TPJ.

Our approach provides insights into the neurobiological mechanisms underlying implicit racial bias and might inform future strategies or interventions with the goal to mitigate elevated implicit racial bias levels. With recent advances in the field of brain stimulation techniques that can non-invasively and specifically enhance SWA and promote cognitive functions^80,84^, it might also be feasible to promote social cognitive functions.

More broadly, our approach to examine localized aspects of sleep may offer insights into the relationship between sleep characteristics and individual differences in various types of cognitive functioning.

## Data Availability

Relevant data and code are available at https://github.com/ mirjam-studler/IATandSWA upon publication.
